# Association between perceived olfactory dysfunction and all-cause mortality in Chinese adults: A prospective community-based study

**DOI:** 10.7189/jogh.14.04237

**Published:** 2024-11-15

**Authors:** Zhicheng Zhang, Yesong Liu, Yaqi Li, Tingting Geng, Shuohua Chen, Shouling Wu, Xiang Gao

**Affiliations:** 1 School of Public Health, Institute of Nutrition, Zhongshan Hospital, Fudan University, Shanghai, China; 2 Department of Neurology, Kailuan General Hospital, Tangshan, Hebei, China; 3 School of Public Health, Institute of Nutrition, Fudan University, Shanghai, China; 4 Department of Cardiology, Kailuan General Hospital, Tangshan, Hebei, China

## Abstract

**Background:**

Olfactory dysfunction has been suggested to be associated with all-cause mortality. Yet, there is a lack of large-scale cohorts to study this relationship.

**Methods:**

Using data from the Kailuan cohort, we assessed 97 327 Chinese adults for perceived olfactory dysfunction at baseline and gathered mortality data from government records. We used Cox proportional hazards regression models to analyse the risk of all-cause mortality associated with perceived olfactory dysfunction, yielding hazard ratios (HRs) and 95% confidence intervals (CIs) with adjustment for multiple potential confounders.

**Results:**

Over a median follow-up of 6.4-year, we recorded a total of 3903 deaths. Individuals reporting perceived olfactory dysfunction had a higher risk of mortality (adjusted HR = 1.42; 95% CI = 1.02–2.00) compared to those without the dysfunction. In sensitivity analyses, we found similar results after excluding participants with pre-existing obesity, cardiovascular diseases, those younger than 50 years old, individuals diagnosed with cancer or stroke during follow-up, and those who died within two years of follow-up.

**Conclusions:**

Perceived olfactory dysfunction was associated with a high risk of all-cause mortality among Chinese adults. Our study is limited by failure to include a national-representative sample and misclassification of exposure assessment due to use of a subjective question to assess olfactory dysfunction. Further studies with objective are warranted to replicate our findings and understand the underlying mechanisms.

Olfactory dysfunction, characterised by a diminished or altered sense of smell [1], represents a prevalent sensory impairment. Emerging evidence found that olfactory dysfunction was associated with high all-cause mortality rate among older adults, suggesting that olfactory dysfunction could be a marker of aging and degeneration in different organ systems [2–11]. Olfactory dysfunction per se also has great impact on quality of life [12–14]. Studies have shown that inflammation, serum lipid levels, and rapid eye movement (REM) sleep behaviour disorder (RBD) symptoms are closely associated with olfactory dysfunction [15–19]. However, the majority of previous research did not adjust for these potential confounders and these studies were limited by relatively small sample sizes (generally <4000) [2,3,5,7,9–11,20]. To fill this gap, we adjusted for supplement biomarkers (high-sensitivity C-reactive protein (Hs-CRP), high-density lipoprotein cholesterol (HDL-C), low-density lipoprotein cholesterol (LDL-C), triglycerides (TG)) reflecting these potential confounders. We also included uric acid, which is closely associated with cognitive function and cardiovascular disease [21,22], as well as RBD, another well-established prodromal symptom of neurodegenerative diseases [23]. We then prospectively evaluated the association between perceived olfactory dysfunction and the risk of all-cause mortality within two community cohorts of 97 327 Chinese adults, hypothesising that perceived olfactory dysfunction is associated with a high mortality rate.

## METHODS

### Participants

The current analysis was based on two ongoing Chinese cohorts: the Kailuan studies I and II, Tangshan, China. The Kailuan study I (n = 101 510) was established in 2006 and the Kailuan study II (n = 35 865) was established in 2008–2010, as described elsewhere [24]. These studies were followed up biannually, all participants completed standard questionnaires, and underwent physical examinations and laboratory assessments at baseline and each follow-up, details of the Kailuan study were described elsewhere [25,26]. The study received approval from the Ethics Committee of the Kailuan General Hospital, and all participants provided written informed consent. Baseline for our current study was set at 2014–2016, when perceived olfactory dysfunction was assessed through questionnaires among 122 801 individuals who participated the 2014–2016 survey.

### Assessment of perceived olfactory dysfunction

Perceived olfactory dysfunction was assessed via a questionnaire, participants were asked ‘Do you have any problems with sense of smell (for at least 3 months), such as not being able to smell things or things not smelling the way they are supposed to?’ Participant who answered ‘yes’ were classified as having perceived olfactory dysfunction [27].

### Assessment of mortality

Mortality data were collected from local government vital statistics office, where medical certificates of death from local hospitals are routinely gathered. These collected data are subsequently verified by the Data Safety Monitoring Board and the Arbitration Committee, as detailed in previous Kailuan-based studies [4,28–31].

### Assessment of covariates

Participants' information on age, sex, educational level (<500, 500–3000, or ≥3000 renminbi (RMB)/mo), income level (<500, 500–3000, or ≥3000 RMB/mo), occupation (in blue collar occupations/in white collar occupations), alcohol consumption (never, ever drinker), smoking status, snoring status (never/rare, occasionally, frequently, or unknown), and RBD symptom were collected via questionaries at baseline [18,32]. Trained field workers measured the weight and height of the participants, and body mass index (BMI) of each participant was calculated as dividing weight in kilograms by the square of height in meters (kg/m^2^). Overweight was defined as 24≤BMI<28 kg/m^2^, and obesity was defined as BMI≥28 kg/m^2^, according to the Working Group on Obesity in China criteria [33]. Systolic and diastolic blood pressures were measured twice using a mercury sphygmomanometer, and the average of the two readings was used for analysis. Hypertension was defined as systolic blood pressure ≥140 mm of mercury (mm Hg) or diastolic blood pressure ≥90 mm Hg, or use of antihypertensive medications regardless of blood pressure status, or history of diagnosed hypertension. Fasting blood samples were collected to determine concentrations of glucose, triglycerides, LDL-C, HDL-C, uric acid, and Hs-CRP using an autoanalyser (Hitachi 747; Hitachi, Tokyo, Japan) at the central laboratory of Kailuan General Hospital. Diabetes was defined as fasting blood glucose concentration ≥7.0 mmol per litre (mmol/L), or use of oral hypoglycaemic agents or insulin, or history of diagnosed diabetes.

### Statistical analyses

Python (version 3.7.6, 2019; Python Software Foundation, Wilmington, Delaware, United States) and *R* (version 4.2.1, 2023; R Core Team, Vienna, Austria) were used for data processing and statistical analyses, with two-tailed *P* < 0.05 considered statistically significant. Missing values for covariates were filled using the Random Forest method from python scikit-learn package [34]. Person-years for each participant were calculated from baseline to the date of death, or 31 December 2021, whichever came first. Hazard ratios (HRs) and 95% confidence intervals (CIs) of all-cause mortality were estimated using the Cox proportional hazards regression model in the survival R package according to perceived olfactory dysfunction. The hazard models satisfied the proportionality assumption.

We fit three multivariate models: model 1 was adjusted for age and sex; model 2 was further adjusted for educational level (primary school and below, middle school, or college and higher), income level (<500, 500–3000, or ≥3000 RMB/mo), occupations (in blue collar occupations/in white collar occupations), smoking status (never, ever smoker), alcohol consumption (never, ever drinker),and BMI; and model 3 was further adjusted for hypertension (yes, no), diabetes (yes, no), snoring status (never/rare, occasionally, frequently, or unknown) and RBD symptom (never, rare, occasionally), plasma concentrations of triglycerides, LDL-C, HDL-C, uric acid, and log transformed Hs-CRP.

To assess the robustness of our results, we conducted a series of sensitivity analyses. This included the exclusion of participants aged under 50, who are less likely to experience disease-related mortality. Given the possibility that patients may have exhibited symptoms but were not diagnosed with cancer or stroke at baseline, we excluded incident cases of cancer and stroke during the follow-up period. To further minimise the impact of confounders on the observed relationship between perceived olfactory dysfunction and all-cause mortality, we excluded participants who had cardiovascular diseases and obesity at baseline. Finally, we excluded individuals who died within two years after baseline to minimise the possibility of reverse causality.

## RESULTS

**Figure 1:**
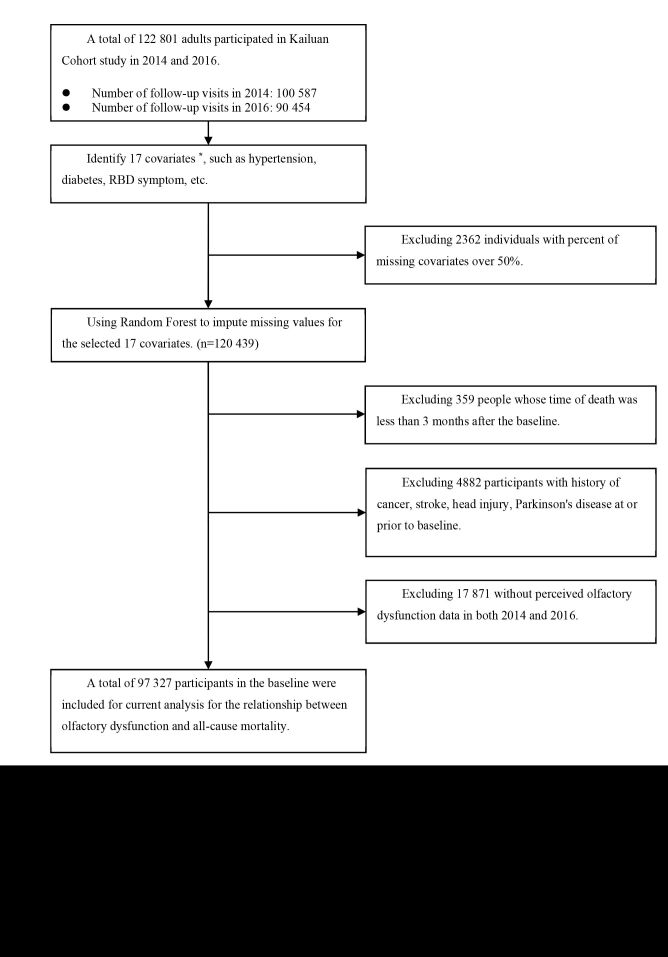
Flowchart of the Kailuan cohort study. Olfactory, all-cause mortality. *Including age and sex, education level (primary school and below, middle school, or college and higher), income level (<500, 500–3000, or ≥3000 renminbi/mo), occupation (in blue collar occupations/in white collar occupations), smoking status (never, ever smoker), alcohol status (never, ever drinker), body mass index, hypertension (no, hypertension), diabetes (no, diabetes), snoring status (never/rare, occasionally, frequently, or unknown), rapid eye movement sleep behaviour disorder symptom (never, rare, occasionally), plasma concentrations of triglycerides, low-density lipoprotein cholesterol, high-density lipoprotein cholesterol, uric acid, and log transformed high-sensitivity C-reactive protein. RBD – Rapid eye movement (REM) sleep behaviour disorder.

After further excluding participants with cancer, stroke, Parkinson disease, a history of head injuries, lacking information on olfactory dysfunction at baseline, missing major covariates data, or death occurring within three months after baseline. A total of 97 327 participants (78 543 men and 18 784 women) were included in the final analysis. A flowchart for the study participants selection is presented in **Figure 1**. Among these participants, 1015 (1.04%) were considered having perceived olfactory dysfunction. Participants with perceived olfactory dysfunction, compared to those without, were more likely to be younger, ever smokers and drinkers, and had higher educational level, lower monthly income, higher prevalence of diabetes, and higher concentrations of BMI, LDL-C, and Hs-CRP. Perceived olfactory dysfunction was also significantly associated with more frequent snoring and RBD symptom. (**Table 1**).

**Table 1:** Baseline characteristics of participants according to olfactory status

Variables	Without perceived olfactory dysfunction	With perceived olfactory dysfunction	*P*-value
N	96 312	1015	
Woman	18 609 (19.3)	175 (17.2)	0.103
Age, year, x̄ (SD)*	51.9 (14.1)	50.4 (12.6)	<0.001
BMI, kg/m^2^, x̄ (SD)	24.9 (3.36)	25.2 (3.53)	0.005
Education, n (%)			
*Primary school and below*	3086 (3.20)	21 (2.07)	0.021
*Middle school*	86 285(89.6)	905 (89.2)	
*College and higher*	6941 (7.21)	89 (8.77)	
Average income (%), RMB/mo			
*<500*	1235 (1.28)	25 (2.46)	0.002
*500-3000*	82 617 (85.8)	872 (85.9)	
*≥3000*	12 460 (12.9)	118 (11.6)	
Occupation, n (%)			
*In white collar occupations*	6582 (6.83)	82 (8.08)	0.134
*In blue collar occupations*	89 730 (93.2)	933 (91.9)	
Snoring, n (%)			
*Never/rare*	80 131 (83.2)	669 (65.9)	<0.001
*Occasionally*	5938 (6.17)	98 (9.66)	
*Frequently*	6396 (6.64)	163 (16.1)	
*Unknown*	3847 (3.99)	85 (8.37)	
RBD symptom†, n (%)			
*Never*	94 467 (98.1)	920 (90.6)	<0.001
*Rare*	1433 (1.49)	75 (7.39)	
*Occasionally*	412 (0.43)	20 (1.97)	
Ever smoker, n (%)	38 203 (39.7)	451 (44.4)	0.002
Ever drinker, n (%)	40 646 (42.2)	505 (49.8)	<0.001
Hypertension, n (%)	40 000 (41.5)	412 (40.6)	0.567
Diabetes, n (%)	12 068 (12.5)	156 (15.4)	0.008
HDL-C, mmol/L, x̄ (SD)	1.37 (0.59)	1.39 (0.48)	0.167
LDL-C, mmol/L, x̄ (SD)	2.92 (1.13)	2.98 (0.81)	0.031
TG, mmol/L, x̄ (SD)	1.79 (2.03)	1.86 (2.36)	0.373
Uric acid, μmol/L, x̄ (SD)	327.6 (91.4)	325.2 (101.4)	0.446
Hs-CRP, mg/L, MD (Q1–Q3)	1.00 (0.46–2.20)	1.20 (0.40–2.40)	0.039

BMI – body mass index, HDL-C – high-density lipoproteins cholesterol, Hs-CRP – high-sensitivity C-reactive protein, LDL-C – low-density lipoproteins cholesterol, MD – median, mmol/L – millimoles per litre, μmol/L – micromoles per litre, RBD – rapid eye movement (REM) sleep behaviour disorder, RMB – renminbi, SD – standard deviation, TG – triglycerides, x̄ – mean

*Adjusted for sex.

**†**The data are sourced from the Kailuan questionnaire, which inquiries about the frequency of nightmares accompanied by limb movement during sleep.

Over a median follow-up of 6.4 years (interquartile range (IQR) = 6.1–7.0 years), we documented 3903 deaths. Presence of perceived olfactory dysfunction was associated with a high risk of all-cause mortality (HR = 1.42; 95% CI = 1.02–2.00), after adjusting for sociodemographic and lifestyle factors, common medical conditions, and indicators of sleep disorder (**Table 2**, Figure S2 in the **Online Supplementary Document**).

**Table 2:** Hazard ratio, 95% confidence interval for risk of all-cause mortality according to olfactory status

Variable	Without perceived olfactory dysfunction	Perceived olfactory dysfunction
No. cases/person-years	3864/600 200	39/4592
Model 1*	1 (ref.)	1.47 (1.05–2.06)
Model 2†	1 (ref.)	1.49 (1.06–2.09)
Model 3‡	1 (ref.)	1.42 (1.02–2.00)

CRP – C-reactive protein, HDL-C – high-density lipoprotein cholesterol, LDL-C – low-density lipoprotein cholesterol

*Adjusted for age and sex.

†Further adjusted for education level (primary school and below, middle school, or college and higher), income level (<500, 500–3000, or >3000 renminbi/mo), occupations (in blue collar occupations/in white collar occupations), smoking status (never, ever smoker), alcohol status (never, ever drinker), body mass index.

‡Further adjusted for hypertension (no, hypertension), diabetes (no, diabetes), snoring status (never/rare, occasionally, frequently, or unknown), rapid eye movement sleep behaviour disorder symptom (never, rare, occasionally), plasma concentrations of triglycerides, LDL-C, HDL-C, uric acid, and log-CRP.

The sensitivity analyses, after excluding individuals with obesity, cardiovascular diseases, those under 50 years old, those who developed cancer or stroke during the follow-up, and those who passed away within the two-year period subsequent to the baseline, generated similar results (**Table 3**). No significant interactions between perceived olfactory dysfunction and potential effect modifiers (age, sex, BMI, hypertension, diabetes, smoking status, and alcohol consumption) were observed (Figure S1 in the **Online Supplementary Document**). 

**Table 3:** Sensitivity analyses for risk of all-cause mortality according to olfactory status*

Variables	Without perceived olfactory dysfunction	Perceived olfactory dysfunction	HRs (95% CI)
	**No. cases/person-years**	**No. cases/person-years**	
Excluding 41 133 participants whose age ≤50	3548 / 334 039	35 / 2307	1.48 (1.04–2.10)
Excluding 1657 participants with cancer (incident)	3182 / 591 720	30 / 4522	1.45 (1.00–2.10)
Excluding 2606 participants with stroke (incident)	3497 / 584 840	39 / 4464	1.56 (1.11–2.18)
Excluding 2584 participants with MI or HF (baseline and incident)	3388 / 585 555	35 / 4453	1.51 (1.06–2.17)
Excluding 19 096 participants with obesity (baseline and incident)†	3204 / 482 250	33 / 3566	1.48 (1.02–2.15)
Excluding 881 death within 24 mo	2990 / 599 153	32 / 4584	1.48 (1.01–2.16)
Without data imputation‡	3830 / 594 889	39 / 4543	1.44 (1.03–2.02)

BMI – body mass index, CI – confidence interval, CRP – C-reactive protein, HDL-C – high-density lipoprotein cholesterol, HF – heart failure, HR – hazard ratio, LDL-C – low-density lipoprotein cholesterol, MI – myocardial infarction

*Adjusted for age, sex, education level (primary school and below, middle school, or college and higher), income level (<500, 500–3000, or >3000 renminbi/mo), occupations (in blue collar occupations/in white collar occupations), smoking status (never, ever smoker), alcohol status (never, ever drinker), body mass index, hypertension (no, hypertension), diabetes (no, diabetes), snoring status (never/rare, occasionally, frequently, or unknown), rapid eye movement sleep behaviour disorder symptom (never, rare, occasionally), plasma concentrations of triglycerides, LDL-C, HDL-C, uric acid, and log-CRP.

†Obesity was defined as BMI≥28 kg/m^2^.

‡For categorical variables, missing data were coded as a separate category. For continuous variables, missing data were removed.

## DISCUSSION

In this large-scale prospective, community-based study, we observed that participants with perceived olfactory dysfunction had a 42% higher all-cause mortality risk relative to those without, after adjusting for conventional risk factors for mortality. Excluding those with major chronic diseases did not change the observed association.

Our results were consistent with previous studies in which an association between olfactory dysfunction and high mortality risk were examined in older adults, despite differences across studies in demographic characteristics of the study population, methods of olfactory assessment, and follow-up duration [2,3,5–11,20]. Most previous studies utilised objective olfactory assessment tests, such the Brief Smell Identification Test, excepted for two studies which utilised self-reported measures of olfaction [2,20]. A study including 1774 adults aged 40 to 90 years [20], for example, reported that the participants who rated their olfactory sensitivity as worse than normal had an approximately 30% higher risk of mortality over ten years compared to those reporting normal olfactory function (adjusted HR = 1.31; 95% CI = 1.06–1.62). In contrast, another cohort including 3503 individuals aged 65 or older failed to find association between self-reported olfactory dysfunction and mortality after five years of follow-up (adjusted HR = 1.12; 95% CI = 0.68–1.84) [2]. None of the previous studies, however, have explored the relationship between olfactory dysfunction and mortality within a large sample size.

Several factors may contribute to the observed association between olfactory dysfunction and mortality. Olfactory dysfunction has been observed to serve as an early warning sign of mild cognitive impairment and neurodegenerative disorders, such as Parkinson disease [35–37], and its severity often corresponded to levels of dementia [38]. This dysfunction was also associated with certain biomarkers, like the apolipoprotein E (APOE) ε4 allele, tau protein, and amyloid-β deposits, which were implicated in adverse health outcomes such as cardiovascular diseases, and Alzheimer’s disease [38,39]. Olfactory dysfunction was also related to depression, likely due to hippocampal pathway damage, which may increase the risk of major chronic diseases and mortality [40–42]. Other pathways, such as vagus nerve dysfunction, could also underly the association between olfactory dysfunction and mortality as vagal effects on olfaction were demonstrated in both animal and human studies [43,44]. For example, obesity was found to suppress stimulation of the vagus-insula-orbitofrontal cortex pathway, thus reducing olfactory abilities [45]. Cancer treatments, such as Radiotherapy (RT, or RT-chemo, or RT-monoclonal antibodies), were also found to impair olfactory function [46]. However, we adjust for major chronic conditions in our model and further analyses excluding individuals with cancer and other major chronic diseases generated similar significant results, suggesting that other pathways could be also involved. Further studies are warranted to understand the underlying mechanisms.

### Strengths and limitations

The strengths of our study include rigorous data collection for a large number of potential confounders among participants, and its prospective study design. However, our study has several limitations. First, olfactory dysfunction was self-reported, which inevitably introduces misclassification bias and could lead the association toward to none. However, the large sample size of our study partially mitigates this issue, suggesting that the use of questionnaires could be a cost-effective method in large population-based studies. Second, the imbalance between the men-to-women ratio may limited our statistical power to detect small-to-modest sex differences. Third, we did not collect data on cognitive function, depression, Alzheimer’s disease, and chronic obstructive pulmonary disease, leaving residual confounding [47–51]. However, excluding individuals with several major chronic conditions (e.g. obesity and cardiovascular diseases) which were well-established risk factors for depression and dementia, did not change the results. Fourth, as all participants were from the Kailuan community, they may not be representative of the general Chinese population and limits the generalisability. Finally, the absence of data on specific causes of mortality in our study precluded further analysing potential mechanisms underlying the link between olfaction and mortality.

## CONCLUSIONS

In this large-scale community-based prospective study, we observed that presence of perceived olfactory dysfunction was associated with high all-cause mortality rate among Chinese adults. Future studies employing objective assessment of olfactory function, with specific measures to evaluate different aspect of olfactory dysfunction, and with cause-specific mortality data collected, to further investigate this observed association is warranted.

## Additional material


Online Supplementary Document
