# Supplementary material for: Association between perceived olfactory dysfunction and all-cause mortality in Chinese adults: A prospective community-based study
Source: J Glob Health. 2024 Nov 15;14:04237. doi: 10.7189/jogh.14.04237 (PMC11565469; doi:10.7189/jogh.14.04237)
Supplement: Online Supplementary Document [file jogh-14-04237-s001.pdf]

Group	Label	Sample Size	Normal (Death, n)	Dysfunction (Death, n)	HR	95% CI	P-interaction
**Age**							0.92
	<60	64,728	921	15	1.54	(0.82, 2.57)	
	≥60	32,599	2,943	24	1.42	(0.94, 2.17)	
**Sex**							0.26
	Men	78,543	3,490	37	1.49	(1.06, 2.11)	
	Women	18,784	374	2	0.56	(0.08, 4.00)	
**BMI**							0.56
	<24	39,098	1,644	13	1.62	(0.92, 2.96)	
	≥24	58,229	2,220	26	1.37	(0.90, 2.09)	
**Hypertension**							0.43
	No	56,915	1,304	11	1.11	(0.53, 2.34)	
	Yes	40,412	2,560	28	1.56	(1.06, 2.28)	
**Diabetes**							0.52
	No	85,103	2,895	26	1.57	(1.03, 2.39)	
	Yes	12,224	969	13	1.27	(0.72, 2.24)	
**Smoking status**							0.25
	Never	58,673	2,484	23	1.18	(0.71, 1.97)	
	Ever smoker	38,654	1,380	16	1.72	(1.09, 2.71)	
**Alcohol intake**							0.24
	Never	56,176	2,522	18	1.18	(0.72, 1.93)	
	Ever drinker	41,151	1,342	21	1.72	(1.08, 2.74)	

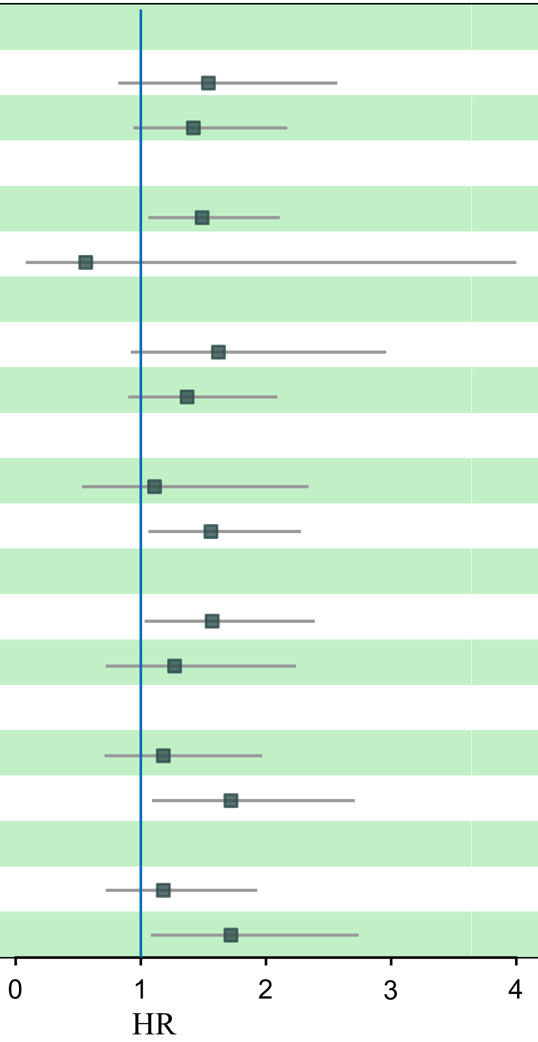

Figure S1. Subgroup analyses for hazard ratios (confidence intervals) of all-cause mortality according to olfactory status [a]

[a] Adjusted for age and sex, education level (primary school and below, middle school, or college and higher), income level (<500, 500–3,000, or ≥ 3,000 renminbi [RMB]/mo), occupations (in blue collar occupations/in white collar occupations), smoking status (never, ever smoker), alcohol status (never, ever drinker), BMI, hypertension (no, hypertension), diabetes (no, diabetes), snoring status (never/rare, occasionally, frequently, or unknown), Rapid Eye Movement (REM) sleep behavior disorder symptom(never, rare, occasionally), plasma concentrations of triglycerides, low-density lipoprotein cholesterol, high-density lipoprotein cholesterol, uric acid, and log transformed high-sensitivity C-reactive protein.

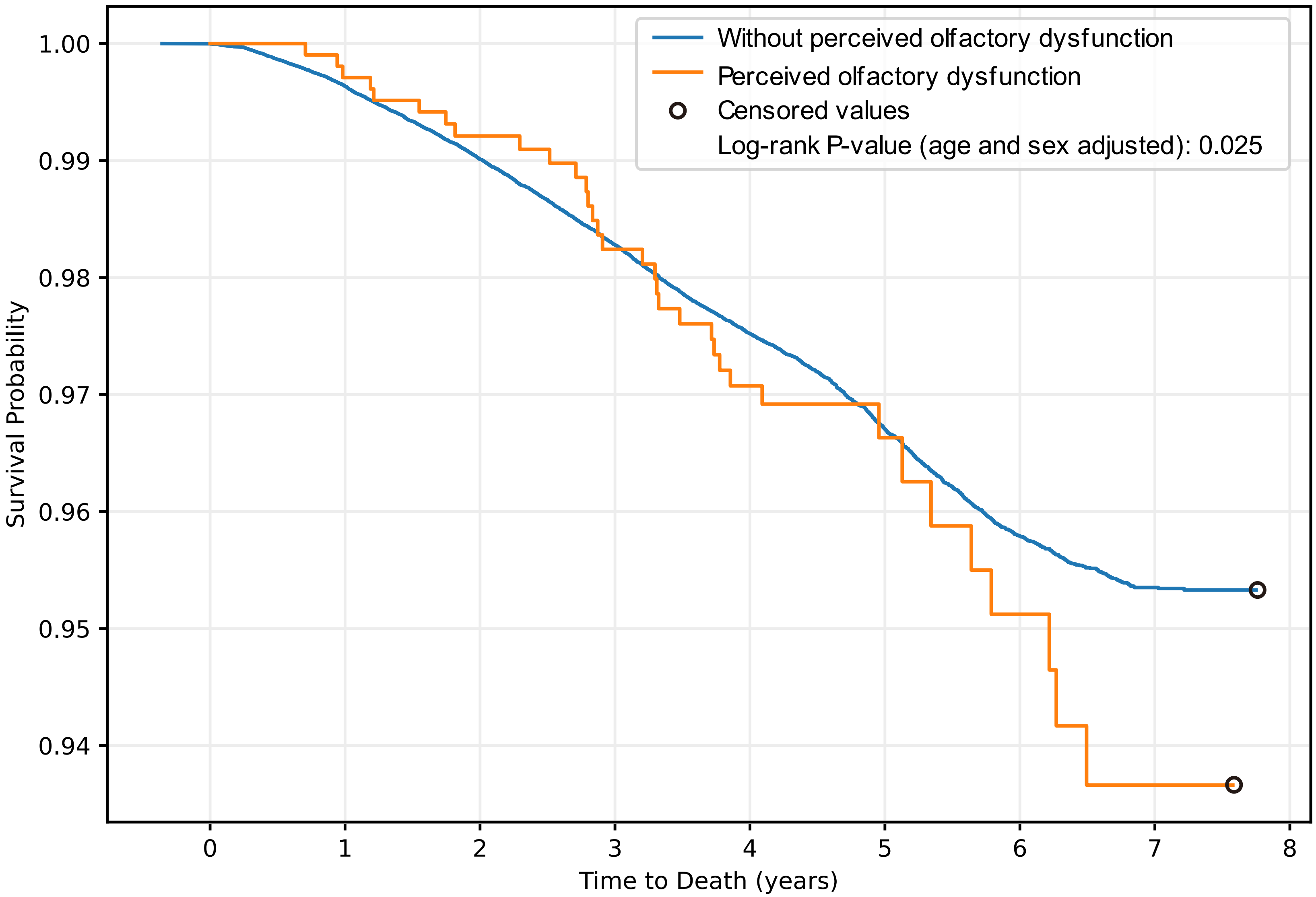

Figure S2. Kaplan-Meier analysis of all-cause mortality according to olfactory status
